# Discussion in the Royal Medical and Chirurgical Society, on the Hypodermic Administration of Certain Medicines

**Published:** 1865-09

**Authors:** 


### SELECTED.

### DISCUSSION IN THE ROYAL MEDICAL AND CHIRURGICAL SOCIETY, ON THE HYPODERMIC ADMINISTRATION OF CERTAIN MEDICINES.

Charles Hunter, Esq., brought forward the results of his investigations into the effects of medicines when subcutaneously injected. It is now six years since Mr. Hunter proposed the injection of Medicines into the cellular tissue with their general therapeutic object in view. In the case of medicines thus injected for general affects, he called the method the "hypodermic," to distinguish it from the endermic, and from the local injection of Wood. From the endermic method, which term is often and erroneously applied to the hydermic, it differs much; the endermic is a superficial application, which must be uncertain in its action, which may act powerfully and dangerously, or prove wholly useless. The hypodermic differs from the "method of Wood." The latter plan has for its object the local treatment of a local affection. The injection was supposed by Dr. Wood to be efficacious simply through the localization. Theoretically this method must be limited in its sphere of action to neuralgia or sciatica: to those cases alone accesssible to the point of the injecting syringe. Mr. Hunter, in advancing the hypodermic method, maintained that localization of the injection in neuralgic cases was theoretically wrong and practically unnecessary. In 1858 and 1859 he advanced the following propositions:—1. That equal effect followed distant and local injections in neuralgic cases. 2. That by distant injections the ill-effects of repeated localization were avoided, such as local irritation, thickening of the skin, abscess, &c. 3. That diseases can be treated with benefit and curatively by this plan which are neither local nor neuralgic, and which have failed to receive benefit from other modes of medicinal administration. Mr. Hunter is inclined to think that the sickness which used rather frequently to follow the localization of the injection, and which is dreaded as an evil attending subcutaneous puncture, is in part due to the localization of the painful part, as he has hardly found sickness occur at all in his experience of the last few years. The first cases were published in 1859, on which occasion Mr. Hunter proved that diseases affecting the nervous system generally could be treated with benefit by the subcutaneous injection; the cases were serious ones, which had resisted other treatment, and were chiefly cases of insomnia and exhaustion from mania, delirium tremens, tetanus, etc. They exemplified the proposition that, "by the introduction of narcotics into the cellular membrane of the body, we have a mode of attacking and subduing cerebral excitability more rapid, more certain, and more sure in action than by the stomachic doses or narcotics." Cases of spinal irritability and excitement were then treated, and with benefit, in cases in which stomachic doses had failed to relieve. Instances were given of tetanus, chorea, epilepsy, etc. The hypodermic method is considered by the author superior to the stomachic, rectal, and endermic modes of medicinal administration, in emergent cases, in which the indications are for anodynes, antispasmodics, hypnotics, and nerve-tonics. And he has found greater and more permanent benefit to accrue from this mode of treatment than from the stomachic use of morphia, atropia, codeia, and other alkaloids. In many diseases there can be no certainty about the stomachic dose. In delirium tremens, for instance, the pill, the draught, or powder, may lie in the stomach undigested; it may be vomited; it may be absorbed, partly or wholly, and, if the latter, so slowly as to do no real good. In the meanwhile the life of the patient is at stake, and death from exhaustion may occur before that sleep which would save the patient can be procured. The investigations of the author led him to believe the hypodermic administration of any alkaloid for the above-mentioned purposes to be more beneficial than the stomachic as regards rapidity of action, certainty of effect, purity of effect, greater permanence of effect, avoidance of exhaustion. 1. Rapidity of effect. With morphia sleep can be procured, or delirium quieted, in a few minutes. Atropine will affect the mouth in two or three minutes, and ease the pain of sciatica when injected into the tissue of the arm in five or ten minutes—in less time, in fact, than is usually occupied for absorption into the blood of the remedy from the stomach. 2. Certainty of effect. This, as a rule, ought to follow, for the whole amount (when properly and carefully) injected must be all absorbed and circulated. But by the stomach one can only guess at the amount absorbed, and consequently one has often to repeat the dose to approach a certainty of effect. Enormous doses and quantities of opium may lie in the stomach doing no good in maniacal and other cases, but rather doing harm through the delay their inertness causes, and the uncertainty as to their absorption. In such cases the hypodermic injection of a small dose will often answer at once, while the opium still lies unabsorbed in the stomach. 3. Purity of effect. As injected into the cellular tissue, so is the agent absorbed, and its direct and sure effects manifest themselves upon the system. The same agent, e. g. morphia, given by the mouth will often cause headache, sickness, giddiness, hepatic and bowel constipation, etc. These ill effects, as they do not, as a rule, follow the hypodermic injection, must be due to the different mode of administration; nor need we wonder that a digesting membrane ever being filled with all kinds of pabulum should modify the vegetable alkaloids whilst chemically acting upon other compounds. 4. Greater permanence of effect. The author has had various cases of neuralgia and sciatica which for years resisted the internal administration of opium, morphia, belladonna, strychnia, etc., which cases he has cured with the injection of morphia, atropia, strychnia, or some other alkaloid. The cure has no necessary dependence upon the number of injections. One lady who had suffered much from sciatica had no pain for one year after one injection. Another patient has remained free from neuralgia since 1859 after a single injection. In two cases of sciatica, one a hatter, the other a driver to a florist, three or four years have elapsed since they were treated; there has been no relapse in either: both had walked lame and suffered pain for four or five years. The author attributes the greater benefits thus derived, partly to the first three advantages that he believes the hypodermic method to possess, and partly also to the slight shock that the diseased nerves sustain through the rapid way in which the alkaloid is brought into contact with them. 5. Avoidance of exhaustion. This is an important advantage of the hypodermic method, chiefly due to the rapidity of its action. In the violent spasms of lockjaw, of colic, or even of retention, life may be lost by delay; but even in minor cases, the longer the time that elapses before relief of the spasm or pain obtained, the greater the subsequent exhaustion. Many hours often elapse before any benefit follows the use of stomachic medication, but by the injection of the cellular tissue the desired relief can be obtained in from five to thirty minutes, instead of after many hours. In the case of delirium tremens the continuous muscular excitement—exhausting in proportion to its activity—is often stopped in a very few minutes, and then sleep follows. The tonic effects of certain medicines are more strikingly manifested by the hypodermic than by other modes of administration. So-called tonics may be thus administered with benefit, when they cannot be borne by the mouth. Quinine may, for instance, cause sickness and heacache and fail to do good by the mouth, but greatly benefits by the cellular administration.* Agents not called tonics may act as such when thus employed, and when of the class usually called anodynes, may act more tonically than when given by the mouth. In this paper space must limit to a brief notice of morphia, thus used for its tonic effects. Morphia may be used subcutaneously, not to relieve pain, nor stop spasm, nor as a narcotic; but as a nerve-tonic in cases of great nervous exhaustion, or of irritability or great mental depression. In cases, in fact, in which the brain has been overtaxed, and the mental equilibrium lost, as in some cases of melancholia, accompanied by great and unnecessary anxiety, with insomnia.

*Paper by M. Desvignes, Med. Chir. Soc. Reports.

In mania *a potu* and in delirium tremens the author had even used the puncture, with morphia, so to steady and quiet the mind and nerves of the patient as to enable him in a few minutes to walk to his office and go through his duties. In the drunkard, as in the overtaxed and melancholic case, there is great mental excitement, and worry, anxiety, and insomnia. The stomachic anodyne will constantly fail to produce any effects; but the hypodermic dose, even where it fails to give sleep, will almost invariably remove the anxiety, the restless, and the nervous irritability, which are the states leading to exhaustion, and unfitting him for application to work. Thus administered, morphia has nerve-tonic effects, without the disadvantages that so often attend its use when given by the mouth; for it does not interfere with the liver or the bowels, it does not cloud the brain, occasion loss of appetite, nor cause sickness, with the well-regulated dose. In the mentally overtaxed or the melancholic patient, the night administration will not always cause sleep; it sometimes rather arouses the brain—it may even keep the patient awake, in "a calm state of dreamy doziness," which has the equivalent effect of good sleep the next day; the patient will arise refreshed, mentally stronger, and fit for his day's work. Upon the spinal marrow and its nerves the tonic effects of morphia are more marked when thus administered. The greater permanance of effect when cases have been cured by this plan, and by a comparatively smaller number of doses, seems to indicate that something more than the mere anodyne influence has been at work. A lady, Mrs. R., was subject to neuralgia in 1859. She had been at times relieved by morphia and opium; she was treated once by the morphia puncture, and has not since had neuralgia. Another patient, Mrs. W. W., was subject to repeated attacks of tic doloreux. The morphia injection cured it last year; she has had no return, notwithstanding a trip to St. Petersburg in bad weather. Sciatica is more than simple pain of the nerve; although of long standing, a single puncture with morphia may relieve all the pain, but the stiffness, the lameness, the deficient nutrition, remain for some time. The repetition of the injection will restore power of tone, and if three or four injections are used where these symptoms remain, in long-standing cases, the patients will often say they feel more power return with each injection. Stomachic doses do not produce these tonic effects so strikingly.

The President said the object was one of very great interest, so great that the Council had appointed a committee to investigate it.

Mr. Moore supposed that no new method of treatment had more speedily come into common and deserved repute than that which had been initiated a few years ago by the author. The subcutaneous injection of morphia had from the first been extensively used in the cancer wards of the Middlesex Hospital, and had conferred an amount of relief upon the patients such as no previous method of subduing pain had ever effected. At the same time, he could bear out all that had been said by the author as to the speedy action of the remedy when thus introduced, and as to the common but not invariable exemption of the patients from sickness and loss of appetite after it. He (Mr. Moore) contrasted its effect with the endermic method in a case of cancer involving the nerves of the brachial plexus, and found more relief afforded by Mr. Hunter's plan. He had also employed the hypodermic method in the two arms of another cancer patient, one of which was œdematous, and had found the obstruction of the circulation much delay the action of the morphia. He was therefore disposed to think the general action of the anodyne essential to the relief of pain, as distinguished from its merely local action. But the most important practical use he had made of the hypodermic plan was that of prolonging the chloroform sleep for a long period after an operation. He first employed it more than three years ago, and he believed it had since been adopted by others. He had observed two especial benefits to result from it: one was the prevention of pain, and the other the postponing or obviating of chloroform sickness. When obliged, after removing cancer with the knife, to lay chloride of zinc on the fresh wound, he had injected morphia before the patient awoke from the effects of the chloroform, and had found sleep prolonged many hours, and pain entirely prevented. Sickness had been postponed by it for twelve hours. That it had been entirely prevented he could not assert; but when it did come on it was moderate, and had not occurred till towards the morning following the operation.

Dr. Wynn Williams, whilst agreeing with the author in his chief conclusions, wished to state that he had adopted the subcutaneous injection of morphia since it was first recommended by Alexander Wood, and in his experience local pain was more speedily relieved by injection locally. For instance, when the pain was neuralgic or of the nature of tic, and the source of irritation eccentric, the injection had better be made as near as possible to the seat of pain, or, at any rate, in the track of nerves having a similar origin. Where the source of irritation was eccentric, the pain depending on disease of the brain or spinal chord, it would answer as well to inject in any other part. In the first class of cases the injection relieved the local pain before it induced sleep by its general action. As regards permanency of action, he did not feel sure that he had caught the author's meaning—i. e., whether he meant the sleep was more continuous, or that the cure was more permanent. He (Dr. Williams) had observed that the effects of morphia given by subcutaneous injection were much more speedy and passed more rapidly off than when administered by the mouth. When thus given, the dose would be larger and the action more continuous, the absorption of it into the system being more gradual. Another good effect was that, given by injection, opium did not constipate the bowels. It would always induce sleep when given in a sufficiently large dose. In a case of cancer of the rectum, which he had attended with Mr. Curling, four grains of morphia had been injected, and this would produce sleep for six hours, with slight intermissions. When the injection was made over the sacrum, the pain was relieved before sleep was induced; if elsewhere, not until the patient went to sleep.

Mr. Savory said all would agree that the hypodermic method was a valuable means of administering medicines, but he thought that some would hardly be prepared to accept all the conclusions at which the author had arrived. In comparing the introduction of drugs by this means with that by the stomach, it would need accurate and extended research before any general results could be obtained, so much must be allowed to the different action of different substances. For example, with regard to rapidity of effect, prussic acid seemed to pass from the stomach at a rate which could scarcely be exceeded, whereas woorara was hardly absorbed at all through the mucous membrane, and between these came a host of substances exhibiting every variety in this respect. Nor was this due to the previous action of the gastric juice, for some substances, such as strychnia, might be kept in it for a long period without any sensible alteration. But then there is another means of administering medicines and other substances—by the rectum. It was perhaps, not generally known that absorption is, under some conditions, more active from the rectum than from the stomach. Not of solids, for these will remain a long while in the rectum without being dissolved; but when substances are in a state of complete solution, they may pass from the rectum far more quickly than from the stomach. Strychnia, for example, when in solution, will kill more rapidly and in smaller doses, when introduced by the rectum than when taken by the mouth. A fourth of a grain or even less, for instance, when passed into the rectum, will act with greater energy than a grain or more when given by the stomach. No doubt each of these several means is, in its turn, the best—no one can be said to possess a constant or uniform advantage over the others. We have yet to learn under what circumstances and for which remedies this or that method should be chosen; and the subject requires immediate investigation.

Dr. Fuller could bear witness to what the author had stated as to the general effect of injection of morphia. It was quite true that if the injection were made near the seat of pain the relief might be a little more speedy, but we owe to Mr. Hunter the observation that the injection of morphia in any part acted in the same way and to nearly the same extent. This was an important practical point, as the continued introduction at one p rt might lead to abscesses.

Mr. Hulke said he should not like the meeting to break up with the idea that four grains of morphia might be safely injected. He had known patients nearly lost by the injection of two thirds of a grain.

Dr. Wynn Williams explained that in his patient's case the large dose of four grains had been gradually arrived at after two years' treatment. He did not mean in any way to imply that this was a safe dose. Indeed, the dose he generally commenced with was only a quarter of a grain.

Dr. A. P. Stewart could not admit Mr. Moore's experiment on the anasarcous compared with the healthy limb, as conclusive regarding the local and general action ot morphia; for in the anasarcous limb there was a total arrest of absorption, without which there could be no therapeutical action. As to the inconvenience of hypodermic injections, the could not speak so lightly ot them as others did. He had repeatedly seen nausea, vomiting, vertigo, and even mild delirium, following the subcutaneous injection of morphia. As regards the use of quinine, he had on a former occasion made some inquiry as to effects, both local and general, of its hypodermic use, the author of the paper (M. Desvignes) having been silent as to the existence of any inconvenience, and he (Dr.

Stewart) had hoped that Mr. Moore would have given the Society the results of his recent and very interesting experiments. He (Dr. Stewart) had often administered remedies by the rectum. His favorite method of treating intestinal obstructions (ileus) had long been the injection of extract of belladonna in doses of two or three grains. In one case, seven or eight grains had been given by mistake. The absence of any serious symptoms was probably attributable to the great abdominal distension, which prevented absorption taking place to any extent; but in other cases, after repeated smaller doses, the wild and extravagant delirium of poisoning by belladonna had been fully developed. He was not aware of the fact stated by Mr. Savory, that strychnia acted more powerfully when given by the rectum. But such a conclusion was not warranted by the fact of the prompt and powerful action of one-sixth of a grain so administered, for he (Dr. Stewart) never gave by the mouth a larger dose than the fiftieth of a grain to begin with, and never increased it beyond the twenty fifth, and from these doses he obtained very decided results. As regards the general subject of the hypodermic injection of remedies, he thought the Society should suspend judgment until the committee had reported on it.

The President asked if the results ot Mr. Hunter's paper had been drawn from public or private practice, and if patients in private practice objected to the treatment.

Mr. Hunter, in reply to the President, said that since the cases which he had treated at St. George's Hospital, he had drawn his conclusions upon patients of a public charity (the Royal Pimlico Dispensary,) but had also largely used the treatment in private practice. For the most part he found no objection on the part of patients to the use of the plan. A nervous patient might occasionally have objected to it, having heard it called an operation. It was one of the simplest kind, giving hardly any or no pain, and causing no irritation, it the operator attended to simple rules. The puncture was almost painless if made in the outer part of the arm; it was more painful if localized to the tender neuralgic site. In answer to Dr. Williams, the author said he had employed the treatment in neuralgia of centric and eccentric origin. He had not found, taking the cases collectively, that the results were more favorable, if localized, in those of eccentric origin. In incurable neuralgia of centric origin the relief was the same in most cases, wherever injected. Sleep was not at all a necessary result of the morphine puncture; many patients did not sleep from it. The injected alkaloid seemed at once to be engaged in removing the pain or the mental excitement existing at the time of the puncture. Sleep might follow or not, according to the dose. In reply to Dr. Stewart, Dr. Hunter said that sickness and giddiness might certainly occur from the use of the morphia, but such cases were quite the exception. They might result from the injection of a dose too large for the nature of the case, or from some idiosyncrasy of the patient. He had very rarely found sickness to follow the injection in his practice of the last few years (though using the plan daily), since he had injected smaller doses of the alkaloids, and had for the most part ceased to localize. He thought four grains a fatal quantity to inject beneath the human skin, except with the well-accredited understanding that the patient had been accustomed to a much larger dose by the mouth. He had himself injected more than four grains at a time, but the patient had for many years taken by the mouth thirty grains of morphia daily. One grain of morphia was quite the maximum for a first injection, and then only in most urgent cases—as in delirium tremens with great excitement. Half a grain of morphia was a full injecting dose for the treatment of most cases: a much less quantity was often quite sufficient. He quite believed in greater permanence of effect by this plan than by the stomachic dose. Atropine, for instance, had often failed, when given daily by the mouth, to do more than palliate; but the same dose (a thirtieth of a grain) by the cellular tissue would perhaps succeed if administered once or twice. With regard to Mr. Savory's observations, the author wished him to understand that the treatment as proposed by him was only for *emergent* cases; as for those requiring speedy relief from pain, or for those in which medicines failed in their effect as usually given. He could cite one or more instances of each class of diseases mentioned in the diagram in which stomachic treatment had previously failed to relieve the patient; and in most cases the same alkaloid was used hypodermically with permanent effect. Mr. Hunter readily allowed that very speedy action followed the rectal administration of some medicines. It might be even quicker than the hypodermic method; but he considered it a less certain and less liable mode of introducing the medicine. It was one which could not always be recommended for cases of the description for which the hypodermic plan seemed to be well adapted.

